# Rhabdomyolysis Associations with Antibiotics: A Pharmacovigilance Study of the FDA Adverse Event Reporting System (FAERS)

**DOI:** 10.7150/ijms.38605

**Published:** 2019-10-15

**Authors:** Chengwen Teng, Courtney Baus, James P. Wilson, Christopher R. Frei

**Affiliations:** 1 Pharmacotherapy Division, College of Pharmacy, The University of Texas at Austin, San Antonio, TX, USA; 2 Pharmacotherapy Education and Research Center, Long School of Medicine, The University of Texas Health Science Center at San Antonio, San Antonio, TX, USA; 3 Health Outcomes Division, College of Pharmacy, The University of Texas at Austin, Austin, TX, USA; 4 South Texas Veterans Health Care System, San Antonio, TX, USA; 5 University Health System, San Antonio, TX, USA

**Keywords:** rhabdomyolysis, adverse drug events, antibiotics, antimicrobial stewardship

## Abstract

**Introduction:** Daptomycin, macrolides, trimethoprim-sulfamethoxazole, linezolid, fluoroquinolones, and cefdinir are known to be associated with rhabdomyolysis. Other antibiotics may also lead to rhabdomyolysis, but no study has systemically compared rhabdomyolysis associations for many available antibiotics.

**Objectives:** The objective of this study was to evaluate the association between rhabdomyolysis and many available antibiotics using the FDA Adverse Event Report System (FAERS).

**Methods:** FAERS reports from January 1, 2004 to December 31, 2017 were included in the study. The Medical Dictionary for Regulatory Activities (MedDRA) was used to identify rhabdomyolysis cases. Reporting Odds Ratios (RORs) and corresponding 95% confidence intervals (95%CI) for the association between antibiotics and rhabdomyolysis were calculated. An association was considered statistically significant when the lower limit of the 95%CI was greater than 1.0.

**Results:** A total of 2,334,959 reports (including 7,685 rhabdomyolysis reports) were considered, after inclusion criteria were applied. Daptomycin had the greatest proportion of rhabdomyolysis reports, representing 5.5% of all daptomycin reports. Statistically significant rhabdomyolysis RORs (95% CI) for antibiotics were (in descending order): daptomycin 17.94 (14.08-22.85), cefditoren 8.61 (3.54-20.94), cefaclor 7.16 (2.28-22.49), erythromycin 5.93 (3.17-11.10), norfloxacin 4.50 (1.44-14.07), clarithromycin 3.95 (2.77-5.64), meropenem 3.19 (1.51-6.72), azithromycin 2.94 (1.96-4.39), cefdinir 2.84 (1.06-7.62), piperacillin-tazobactam 2.61 (1.48-4.61), trimethoprim-sulfamethoxazole 2.53 (1.52-4.21), linezolid 2.49 (1.47-4.21), ciprofloxacin 2.10 (1.51-2.92).

**Conclusions:** This study confirms prior evidence for rhabdomyolysis associations with daptomycin, macrolides, trimethoprim-sulfamethoxazole, linezolid, fluoroquinolones, and cefdinir. This study also identifies previously unknown rhabdomyolysis associations with meropenem, cefditoren, cefaclor, and piperacillin-tazobactam.

## Introduction

Rhabdomyolysis is a clinical syndrome of skeletal muscle injury associated with myoglobinuria, electrolyte abnormalities, and acute kidney injury 1. The severity of rhabdomyolysis can range from an asymptomatic elevation in creatinine kinase (CK) to severe life-threatening symptoms associated with high CK levels and renal failure. The most common presenting symptoms include myalgia, weakness, and tea-colored urine.

Some antibiotics have been shown to play a role in developing rhabdomyolysis. Multiple case reports have shown an association between daptomycin and rhabdomyolysis 2-4. The use of macrolides alone or in combination with statins was reported to be related to rhabdomyolysis 5,6. A review of 53 cases showed rhabdomyolysis associated with concomitant use of statins and azithromycin 7. Additionally, case reports indicated that trimethoprim-sulfamethoxazole induced rhabdomyolysis occurred in both immunocompetent and immunocompromised patients 8,9. There have been case reports linking the use of linezolid to serious cases of rhabdomyolysis 10,11. Fluoroquinolones have also been shown to be associated with rhabdomyolysis in case reports when fluoroquinolones were used alone or in combination with statins 12,13. Rhabdomyolysis is listed as an adverse drug reaction (ADR) of cefdinir in its United States Food and Drug Administration (FDA) label 14. Rhabdomyolysis was also associated with certain medications, such as statins 15.

Antibiotics are frequently prescribed home medications, so it is imperative to know which classes and specific agents within classes are more likely to be associated with rhabdomyolysis. Although some antibiotics are known to be associated with rhabdomyolysis, no study has systematically compared rhabdomyolysis associations for common antibiotics. The objective of this study was to evaluate the association between rhabdomyolysis and common antibiotics using the FDA Adverse Event Report System (FAERS).

## Methods

### Data Source

FAERS is a publicly available database, which is composed of adverse event reports that were submitted to the FDA 16. FAERS data contain drug information (drug name, active ingredient, route of administration, the drug's reported role in the event) and reaction information. Each report has a primary suspected drug with one or more ADRs and may include other drugs taken by the patient.

### Study Design

The methods of data mining were previously used in other published FAERS studies 17-19. FAERS data from January 1, 2004 to December 31, 2017 were included in the study. Some reports were submitted to FDA multiple times with updated information. Therefore, duplicate reports were removed by case number, with only the most recently submitted version included in the study. Another step of removing duplicate reports was performed by matching age, sex, event date, and reporter country.

### Drug Exposure Definition

Each antibiotic was identified in FAERS by generic and brand names listed in the Drugs@FDA Database 17-20. Drugs with a reported role coded as “PS” (Primary Suspect Drug) were evaluated for inclusion. Antibiotics with less than three ADR reports were excluded from data analysis 21.

### Adverse Drug Reaction Definition

FAERS defines ADRs using Preferred Terms (PT) from the Medical Dictionary for Regulatory Activities (MedDRA) 22. Preferred Term “Rhabdomyolysis” was used to identify rhabdomyolysis cases. Standardised MedDRA Queries (SMQs) are groupings of MedDRA terms, usually at the PT level, which relate to an adverse drug reaction. Hepatic disorders (SMQ) was used to identify cases with hepatic disorders. Acute renal failure (SMQ) and the term “Renal failure acute” were used to identify acute kidney injury cases.

### Statistical Analysis

A disproportionality analysis was conducted by computing Reporting Odds Ratios (ROR) and corresponding 95% confidence intervals (95%CI) for the association between rhabdomyolysis and each antibiotic class or individual antibiotic or each statin 23. ROR was calculated as the ratio of the odds of reporting rhabdomyolysis versus all other ADRs for a given drug, compared with this reporting odds for all other drugs present in FAERS 23. An association was considered to be statistically significant if the lower limit of 95%CI was above 1.0 23. A higher ROR suggested a stronger association between the antibiotic and rhabdomyolysis. An adjusted ROR was calculated after removing reports of potentially confounding statins from the data analysis. These drugs include atorvastatin, fluvastatin, lovastatin, pitavastatin, pravastatin, rosuvastatin, and simvastatin. Rhabdomyolysis RORs for meropenem reports with hepatic disorders and without hepatic disorders were calculated. Rhabdomyolysis RORs for piperacillin-tazobactam reports with acute kidney injury and without acute kidney injury were computed. Data analysis was performed using Microsoft Access 2016, Microsoft Excel 2016 (Microsoft Corporation, Redmond, WA), SAS 9.4, and JMP Pro 13.2.1 (SAS Institute, Cary, NC).

## Results

**Figure 1:**
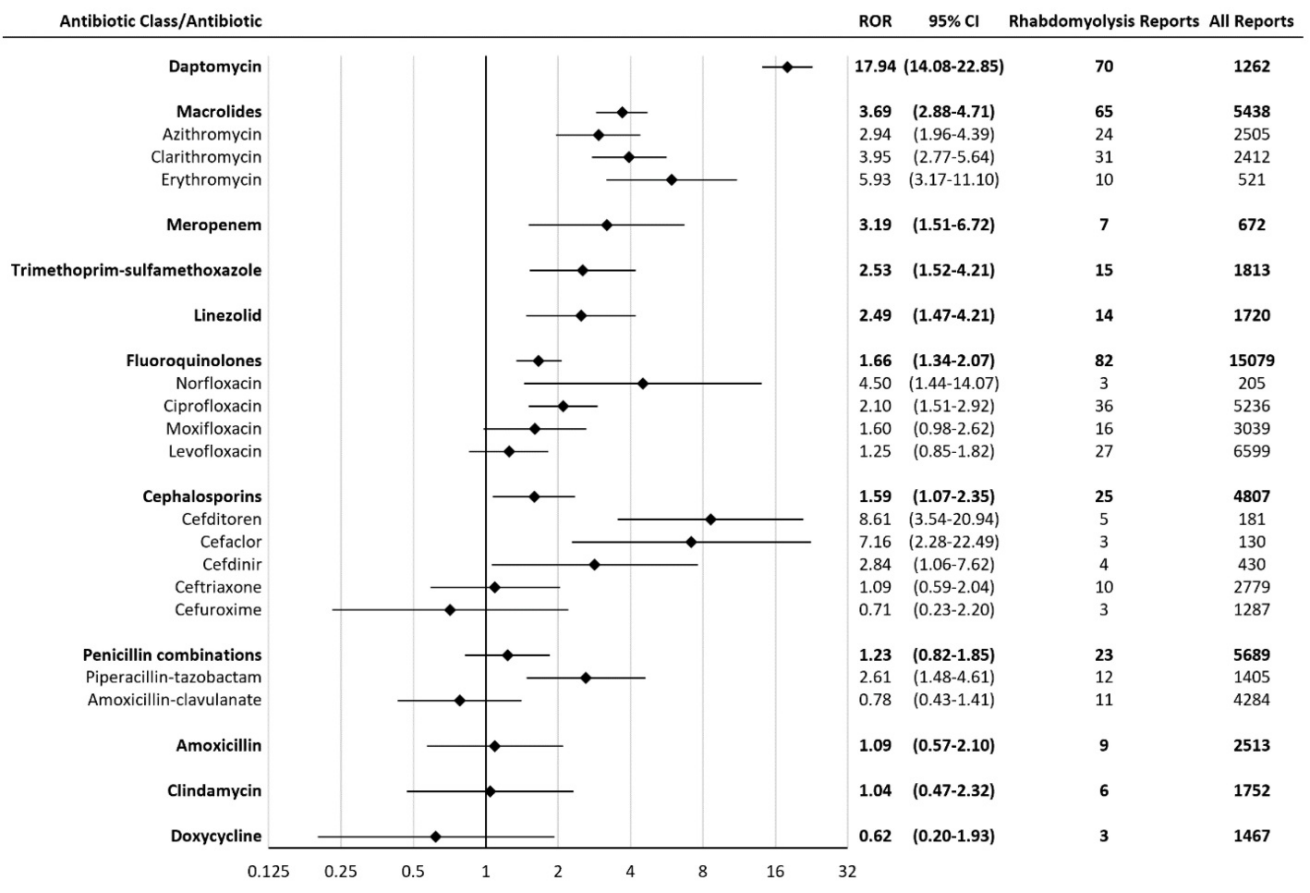
Reporting Odds Ratios (RORs) for rhabdomyolysis with antibiotics. CI = confidence interval.

A total of 2,334,959 reports (including 7,685 rhabdomyolysis reports) were considered, after inclusion criteria were applied. Daptomycin had the greatest proportion of rhabdomyolysis reports, representing 5.5% of all daptomycin reports. Statistically significant rhabdomyolysis RORs (95% CI) for antibiotics were (in descending order): daptomycin 17.94 (14.08-22.85), cefditoren 8.61 (3.54-20.94), cefaclor 7.16 (2.28-22.49), erythromycin 5.93 (3.17-11.10), norfloxacin 4.50 (1.44-14.07), clarithromycin 3.95 (2.77-5.64), meropenem 3.19 (1.51-6.72), azithromycin 2.94 (1.96-4.39), cefdinir 2.84 (1.06-7.62), piperacillin-tazobactam 2.61 (1.48-4.61), trimethoprim-sulfamethoxazole 2.53 (1.52-4.21), linezolid 2.49 (1.47-4.21), ciprofloxacin 2.10 (1.51-2.92) (Figure 1). Meropenem had seven rhabdomyolysis reports, including four reports with hepatic disorders. Rhabdomyolysis RORs (95% CI) for meropenem reports with hepatic disorders and without hepatic disorders were 9.77 (3.61-26.46) and 1.68 (0.54-5.23), respectively. Cefditoren had five rhabdomyolysis reports, including one report of 5-year-old girl with decreased carnitine level. Cefaclor had three rhabdomyolysis reports, including two reports on concomitant Nonsteroidal Anti-Inflammatory Drugs (NSAIDS), which were indomethacin and flurbiprofen. Piperacillin-tazobactam had twelve rhabdomyolysis reports, including three reports with acute kidney injury. Rhabdomyolysis RORs (95% CI) for piperacillin-tazobactam reports with acute kidney injury and without acute kidney injury were 4.91 (1.57-15.37) and 2.26 (1.17-4.35), respectively.

**Figure 2:**
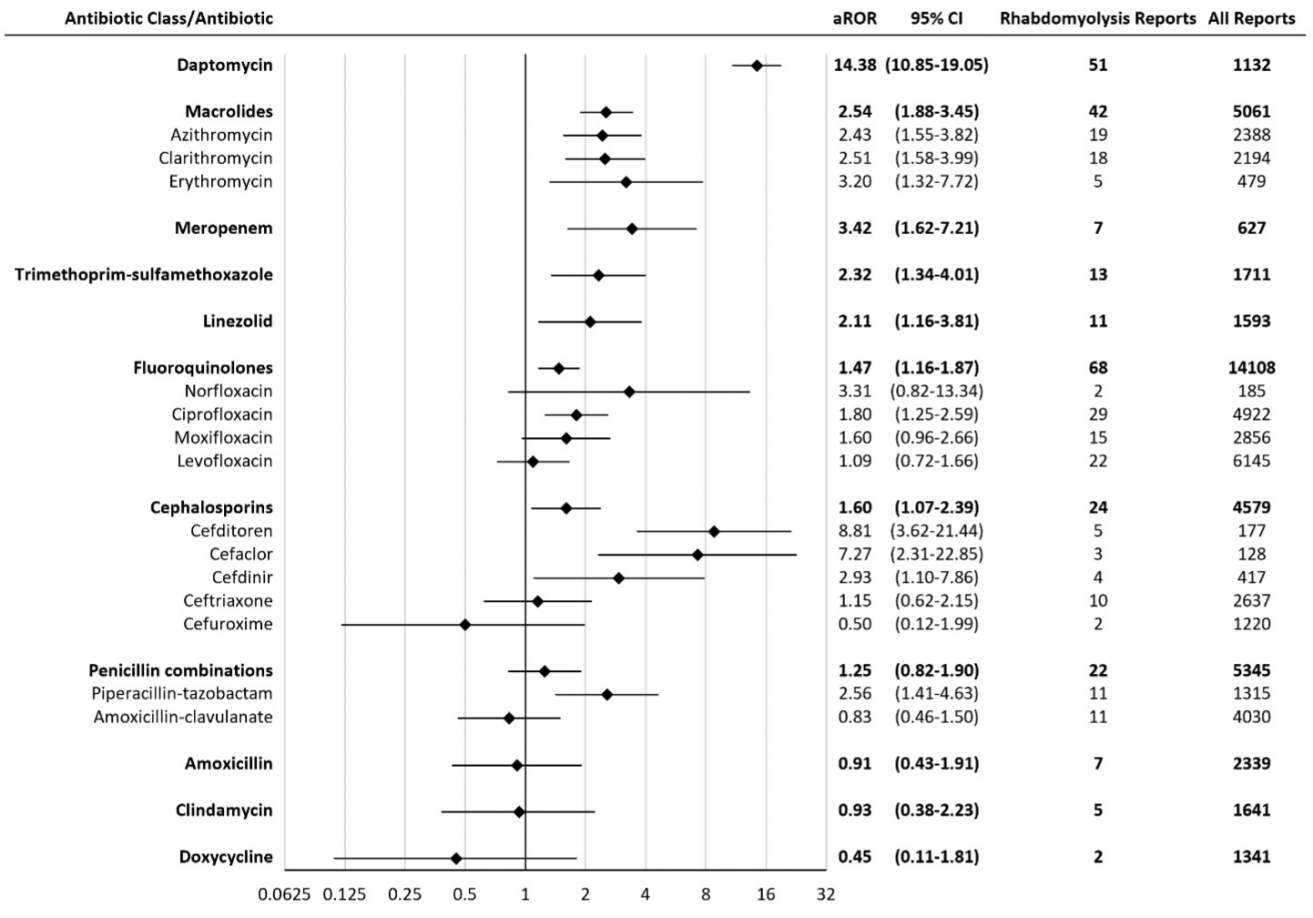
Adjusted Reporting Odds Ratios (aRORs) for rhabdomyolysis with antibiotics. CI = confidence interval. All statins were significantly associated with rhabdomyolysis. Rhabdomyolysis RORs (95% CI) for statins range from atorvastatin 15.09 (13.77-16.53) to simvastatin 76.29 (70.92-82.06).

An adjusted ROR was calculated after removing reports of concomitant statins. The adjusted RORs for agents with a significant association with rhabdomyolysis were: daptomycin 14.38 (10.85-19.05), cefditoren 8.81 (3.62-21.44), cefaclor 7.27 (2.31-22.85), meropenem 3.42 (1.62-7.21), erythromycin 3.20 (1.32-7.72), cefdinir 2.93 (1.10-7.86), piperacillin-tazobactam 2.56 (1.41-4.63), clarithromycin 2.51 (1.58-3.99), azithromycin 2.43 (1.55-3.82), trimethoprim-sulfamethoxazole 2.32 (1.34-4.01), linezolid 2.11 (1.16-3.81), ciprofloxacin 1.80 (1.25-2.59) (Figure 2). The ROR association rank had little change when adjusted to exclude statins.

All statins were significantly associated with rhabdomyolysis. RORs (95% CI) for statins were (in descending order): simvastatin 76.29 (70.92-82.06), lovastatin 50.95 (34.59-75.05), pitavastatin 38.92 (27.86-54.35), fluvastatin 24.51 (16.89-35.58), pravastatin 23.66 (17.83-31.40), rosuvastatin 22.94 (20.75-25.37), atorvastatin 15.09 (13.77-16.53).

## Discussion

Our results demonstrated significant rhabdomyolysis associations (from strongest to weakest) with daptomycin, cefditoren, cefaclor, erythromycin, norfloxacin, clarithromycin, meropenem, azithromycin, cefdinir, piperacillin-tazobactam, trimethoprim-sulfamethoxazole, linezolid, and ciprofloxacin. Our results confirmed previously known rhabdomyolysis associations with daptomycin, macrolides, trimethoprim-sulfamethoxazole, linezolid, fluoroquinolones, and cefdinir 2-14. Meropenem, cefditoren, cefaclor, and piperacillin-tazobactam were found to be associated with rhabdomyolysis, which were not reported in the literature. In a case report, meropenem induced prolonged severe hypokalemia and hypomagnesaemia, leading to chronic muscle weakness 24. Meropenem-induced hypokalemia and hypomagnesaemia might play a role in meropenem-induced muscle injury and rhabdomyolysis. In our study, four out of seven meropenem-associated rhabdomyolysis reports had hepatic disorders. Rhabdomyolysis ROR for meropenem reports with hepatic disorders was higher than those without hepatic disorders. Since meropenem is hepatically metabolized 25, hepatic disorders might be a risk factor for meropenem-induced rhabdomyolysis.

A case report demonstrated that cefditoren pivoxil induced hypocarnitinemia and hypoglycemia in a 6-year-old girl with Fukuyama-type congenital muscular dystrophy 26. In our study, a 5-year-old girl on cefditoren had both rhabdomyolysis and decreased carnitine level. Although there were commonalities between these two cases, more data would be necessary prior to making any conclusions between hypocarnitemia and rhabdomyolysis. In addition, hypocarnitemia may be a sign of Long-Chain 3-Hydroxyacyl-Coenzyme A Dehydrogenase (LCHAD) deficiency which can cause rhabdomyolysis. In this respect, it is possible that the cefditoren did not cause the rhabdomyolysis, but these patients had underlying illness that caused them to have rhabdomyolysis.

There were three cefaclor-associated rhabdomyolysis reports in our study. Two of these reports had concomitant NSAIDS. NSAIDS are known to be associated with acute kidney injury 27 and acute kidney injury might increase the cefaclor exposure which led to rhabdomyolysis. However, since acute kidney injury was used to diagnose rhabdomyolysis, the association between rhabdomyolysis and cefaclor was confounded by acute kidney injury.

In our study, twelve piperacillin-tazobactam reports listed rhabdomyolysis as an ADR. Three of these reports had acute kidney injury as well. Rhabdomyolysis ROR for piperacillin-tazobactam reports with and without acute kidney injury were statistically similar.

## Limitations

A causal relationship between a drug and an ADR cannot be determined by FAERS. Significant bias may occur because of the spontaneous and voluntary reporting of ADRs. Media attention and recent publication of an ADR in the literature might affect the reporting behaviors. The association between a drug and an ADR is confounded by comorbidities and concomitant drugs 19. Despite the limitations, FAERS has a large sample size and is suitable for discovering new and rare drug-ADR associations.

## Conclusions

This study confirms prior evidence for rhabdomyolysis associations with daptomycin, macrolides, trimethoprim-sulfamethoxazole, linezolid, fluoroquinolones, and cefdinir. This study also identifies previously unknown rhabdomyolysis association with meropenem, cefditoren, cefaclor, and piperacillin-tazobactam. Results obtained from FAERS should be interpreted with caution in the context of data limitations. Antibiotic stewardship is needed to prevent rhabdomyolysis and to improve health outcomes. Monitoring of baseline and weekly CK levels for daptomycin and other antibiotics which were associated rhabdomyolysis is warranted.

## Abbreviations

ADR: adverse drug reaction
CK: creatinine kinase
CI: confidence interval
FDA: Food and Drug Administration
FAERS: FDA Adverse Event Reporting System
MedDRA: Medical Dictionary for Regulatory Activities
NSAIDS: Nonsteroidal Anti-Inflammatory Drugs
PT: Preferred Term
ROR: Reporting Odds Ratio
SMQ: Standardised MedDRA Queries
